# Supplementary material for: Effects of high-impact jumping versus resistance exercise on bone mineral content in children and adolescents: a systematic review and meta-analysis
Source: PeerJ. 2025 Jun 30;13:e19616. doi: 10.7717/peerj.19616 (PMC12225634; doi:10.7717/peerj.19616)
Supplement: Supplemental Information 2 [file peerj-13-19616-s002.docx]

Supplemental Figure S1:

**Sensitivity analysis of jumping on femoral neck BMC**

**Sensitivity analysis of resistance exercise on femoral neck BMC**

**Sensitivity analysis of jumping on lumbar spine BMC**

**Sensitivity analysis of resistance exercise on lumbar spine BMC**

**Sensitivity analysis of jumping on whole-body BMC**

**Sensitivity analysis of resistance exercise on whole-body BMC**
