# Supplementary material for: Effects of high-impact jumping versus resistance exercise on bone mineral content in children and adolescents: a systematic review and meta-analysis
Source: PeerJ. 2025 Jun 30;13:e19616. doi: 10.7717/peerj.19616 (PMC12225634; doi:10.7717/peerj.19616)
Supplement: Supplemental Information 4 [file peerj-13-19616-s004.docx]

**PubMed：**

| No. | Search Strategy | Results |
| --- | --- | --- |
| #1 | ((((((("Exercise"[Mesh]) OR "Resistance Training"[Mesh]) OR (sport)) OR (High-impact sports)) OR (Impact sports)) OR (Jump)) OR (physical activity)) OR (training) | 172555 |
| #2 | (((((("Adolescent"[Mesh]) OR (student)) OR (Puberty)) OR (children)) OR (kids)) OR (child)) OR (pediatrics) | 259087 |
| #3 | (((bone) OR (bone health)) OR (Bone mineral content)) OR (Bone mineral density) | 54657 |
| #4 | #1 AND #2 AND #3 | 1724 |
| Restrictions | (clinical trial[Filter] OR controlled clinical trial[Filter] OR randomized controlled trial[Filter]). | |

**The Cochrane Library:**

| No. | Search Strategy | Results |
| --- | --- | --- |
| #1 | MeSH descriptor: [Exercise] explode all trees | 39577 |
| #2 | MeSH descriptor: [Resistance Training] explode all trees | 5874 |
| #3 | Impact sports | 2631 |
| #4 | High-impact sports | 73 |
| #5 | Jump | 4349 |
| #6 | physical activity | 68247 |
| #7 | training | 146930 |
| #8 | sport | 15803 |
| #9 | #1OR#2OR#3OR#4OR#5OR#6OR#7OR#8 | 219035 |
| #10 | MeSH descriptor: [Adolescent] explode all trees | 136093 |
| #11 | student | 32431 |
| #12 | Puberty | 1665 |
| #13 | children | 217570 |
| #14 | kids | 2091 |
| #15 | child | 217570 |
| #16 | pediatrics | 32171 |
| #17 | #10OR#11OR#12OR#13OR#14OR#15OR#16 | 342389 |
| #18 | bone | 77654 |
| #19 | bone health | 15088 |
| #20 | Bone mineral density | 10569 |
| #21 | Bone mineral content | 12779 |
| #22 | #18OR#19OR#20OR#21 | 77654 |
| #23 | #9AND#17AND#22 | 1508 |

**Embase**

| No. | Search Strategy | Results |
| --- | --- | --- |
| #1 | 'exercise'/exp OR 'resistance training'/exp OR 'high impact exercise' OR 'jump' OR 'physical activity'/exp | 1010801 |
| #2 | 'adolescent'/exp OR 'student'/exp OR 'puberty'/exp OR 'child'/exp OR 'pediatrics'/exp | 4998940 |
| #3 | 'bone'/exp OR 'bone health'/de OR 'bone mineral density'/exp OR 'bone mineral content' | 1116896 |
| #4 | #1 AND #2 AND #3 | 3115 |
| Restrictions | ('controlled study'/de OR 'randomized controlled trial'/de) | |

**Web of Science**

| No. | Search Strategy | Results |
| --- | --- | --- |
| #1 | resistance training OR high impact sports OR jump | 1021 |
| #2 | Adolescent OR student OR Puberty OR children OR kids OR child OR pediatrics | 3970824 |
| #3 | bone OR bone health OR Bone mineral density OR Bone mineral content | 1074510 |
| #4 | #1 AND #2 AND #3 | 1021 |
